# Augmented Reality Support for Anterior Decompression and Fusion Using Floating Method for Cervical Ossification of the Posterior Longitudinal Ligament

**DOI:** 10.3390/jcm12082898

**Published:** 2023-04-16

**Authors:** Hiroaki Onuma, Kenichiro Sakai, Yoshiyasu Arai, Ichiro Torigoe, Masaki Tomori, Kyohei Sakaki, Takashi Hirai, Satoru Egawa, Yutaka Kobayashi, Atsushi Okawa, Toshitaka Yoshii

**Affiliations:** 1 Department of Orthopedic Surgery, Saiseikai Kawaguchi General Hospital, 5-11-5 Nishikawaguchi, Kawaguchi-shi 332-8558, Japan; 2 Department of Orthopedic Surgery, Tokyo Medical and Dental University, 1-5-45 Yushima, Bunkyo Ward, Tokyo 113-8519, Japan

**Keywords:** augmented reality, extended reality, navigation system, computed-tomography-based navigation, ossification of the posterior longitudinal ligament, OPLL, anterior cervical spine surgery, anterior decompression and fusion, floating method, selective wedge corpectomy, perioperative complication

## Abstract

Anterior decompression and fusion (ADF) using the floating method for cervical ossification of the posterior longitudinal ligament (OPLL) is an ideal surgical technique, but it has a specific risk of insufficient decompression caused by the impingement of residual ossification. Augmented reality (AR) support is a novel technology that enables the superimposition of images onto the view of a surgical field. AR technology was applied to ADF for cervical OPLL to facilitate intraoperative anatomical orientation and OPLL identification. In total, 14 patients with cervical OPLL underwent ADF with microscopic AR support. The outline of the OPLL and the bilateral vertebral arteries was marked after intraoperative CT, and the reconstructed 3D image data were transferred and linked to the microscope. The AR microscopic view enabled us to visualize the ossification outline, which could not be seen directly in the surgical field, and allowed sufficient decompression of the ossification. Neurological disturbances were improved in all patients. No cases of serious complications, such as major intraoperative bleeding or reoperation due to the postoperative impingement of the floating OPLL, were registered. To our knowledge, this is the first report of the introduction of microscopic AR into ADF using the floating method for cervical OPLL with favorable clinical results.

## 1. Introduction

In recent years, extended reality (XR) technology, which collectively refers to mixed reality (MR), augmented reality (AR), and virtual reality (VR) technologies, has been developing rapidly. XR technology is beginning to be used in social applications such as education and training, work support, and leisure as well as in the medical field. XR technology is finding its place in healthcare, and spine surgery is no exception. In the past few years, computer-aided technology, such as conventional navigation systems, has advanced. In surgery, a surgical imaging support tool is becoming an indispensable technology, and further utilization of XR technology is expected [1,2]. A unique feature or advantage of the XR device is its flexible integration with navigation systems, a beneficial aspect of surgery.

Cervical ossification of the posterior longitudinal ligament (OPLL) is a degenerative spinal disease that causes neurological dysfunction [3,4,5]. In the early stages of the disease, most patients with OPLL show no neurologic symptoms. However, as the OPLL develops in size, the spinal cord and nerve roots become compressed anteriorly, resulting in myelopathy and/or radiculopathy [6]. Surgical procedures for cervical myelopathy caused by OPLL are often chosen based on the OPLL size and the cervical spine alignment [7,8,9,10,11,12,13,14]. Anterior decompression and fusion (ADF) for cervical OPLL is an ideal surgical technique for the direct decompression of the spinal cord in which the ossification is floated or resected [7,8,9,10,11,12,13,14]. It is reported that laminoplasty for patients with cervical OPLL who have OPLL with a ≥50% occupying ratio and/or K-line (−) OPLL may result in poor neurological recovery [10,13,15,16,17]. Therefore, at our institution, we have generally treated those patients with ADF using the floating method [18]. However, because of the high procedural difficulty of ADF using the floating method, it is sometimes difficult to accurately identify the full shape of OPLL, which exists behind the vertebrae, mixed with ligamentous or cartilaginous tissues [9,19]. Therefore, there is a risk of insufficient decompression caused by the impingement of residual ossification. To avoid this, using an intraoperative imaging modality, such as mobile computed tomography, was previously reported to be effective [20,21]. Recently, intraoperative real-time navigation systems have become available for spine surgeries, including ADF, and help surgeons to avoid disorientation during surgery [22].

Conventional three-dimensional (3D) navigation systems have limitations in displaying the positional relationships between the surgical target and the basic structures in the surgical field. This can make it difficult for surgeons to accurately identify the full shape of OPLL and avoid the insufficient decompression caused by the impingement of residual ossification. However, AR support is a novel technology that enables the superimposition of images, such as radiographs and navigation pathways, onto a view of the surgical field [2,23]. In recent years, AR technology has been employed in spine surgery to better understand the anatomical orientation.

Moreover, AR support is highly flexible and can be integrated with navigation systems, making it an ideal technology for spine surgery. AR support can display preoperative imaging data on the patient during surgery, allowing the surgeon to precisely align implants and instruments with the patient’s anatomy [24,25]. AR support can also assist in the identification and localization of anatomical structures, such as nerves and blood vessels, which can be difficult to visualize during surgery [26]. This has led to improved accuracy in surgical procedures and reduced the risk of complications.

In this study, we report our surgical experience of applying AR support to ADF using the floating method. Our results demonstrate that AR support can improve the accuracy of surgery by enabling surgeons to better identify the full shape of OPLL and avoid the insufficient decompression caused by residual ossification.

## 2. Materials and Methods

### 2.1. Study Designs

This study was a retrospective observational study. This study was approved by the institutional review board of the Saiseikai Kawaguchi General Hospital (approval No. 2022-22), and written informed consent was obtained from all participants. All of the surgeries were performed by attending spine surgeons that were certified by the Japanese Orthopaedic Association and the Japanese Society for Spine Surgery and Related Research.

### 2.2. Participants

This study included 14 patients who underwent AR-assisted spinal surgery since March 2021 and 53 patients who underwent spinal surgery without AR support (as a historical control group) before 2021 at Saiseikai Kawaguchi General Hospital and Tokyo Medical and Dental University.

### 2.3. Surgical Technique of ADF with Microscopic AR Support

#### 2.3.1. Surgical Indications of ADF using the Floating Method for Cervical OPLL

At our institution, patients with cervical OPLL who have OPLL with a ≥50% occupying ratio and/or K-line (−) OPLL were generally treated with ADF. In ADF, the anterior OPLL floating technique was utilized in all patients, as described by Yamaura et al. [7,8,9,10,11,12,18]. Intraoperative spinal monitoring was performed in all patients. A radiolucent carbon operating table was used. The patient was placed in a supine position, the patient’s skull was fixed with a carbon Mayfield clamp, and a reference frame was attached to the clamp.

#### 2.3.2. Surgical Approaches to the Cervical Spine and Preparation for Intraoperative Navigation

**Figure 1:**
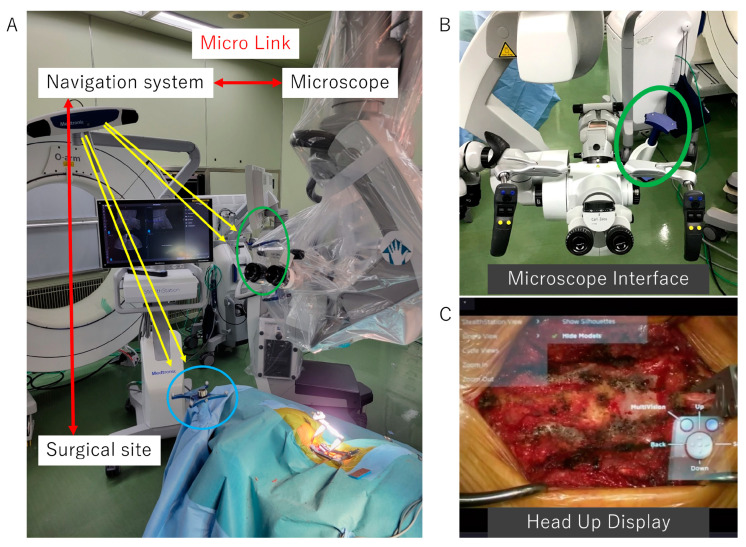
Operating room setting. (**A**) Operating room setting. The navigation camera tracks the microscope interface (green circle) and the reference frame that is attached to the patient (blue circle). (**B**) Microscope interface (green circle) to track its position during surgery. (**C**) AR support information constructed using intraoperative 3D image data is displayed in the surgical field on the head-up display. AR, augmented reality.

A standard Smith–Robinson approach to the cervical spine was employed [27]. After confirmation and exposure of the appropriate vertebral levels, a retractor was placed on the cervical spine. After careful draping, the O-arm mobile intraoperative imaging system (O-arm^TM^ Surgical Imaging System, Medtronic Navigation Inc., Littleton, MA, USA) was used to create an augmented image model in the navigation system (StealthStation^TM^ Surgical Navigation System, Medtronic Navigation Inc., MA, USA) (Figure 1A).

#### 2.3.3. Reconstructed 3D Images of the OPLL Using Anatomical Mapping Software

**Figure 2:**
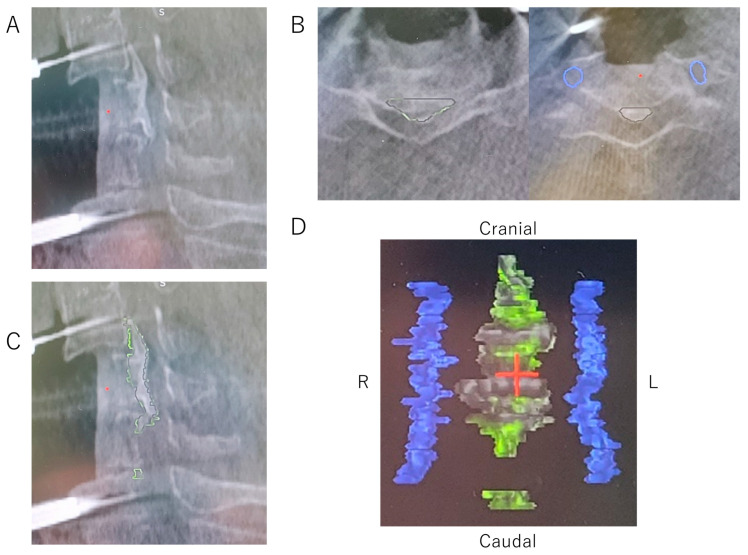
Intraoperative CT images of the OPLL. (**A**) Intraoperative CT sagittal view of the OPLL. (**B**) Outline of ossification (yellow-green line) and blood vessels (blue line) marked on the monitor of the CT axial views. (**C**) Sagittal image of the OPLL, which was reconstructed after marking the outlines of ossification for each axial slice from the cranial end to the caudal end of the OPLL. (**D**) Reconstructed 3D images of the OPLL and bilateral vertebral arteries. CT, computed tomography; OPLL, ossification of the posterior longitudinal ligament.

Three-dimensional objects representing the surgical target were generated by applying anatomical mapping software (Stealth 3D Cranial Software^TM^, Medtronic Navigation Inc., MA, USA). The outline of the ossification was marked on the monitor using axial computed tomography (CT) views taken with the intraoperative O-arm system. The ossification was marked for each axial slice from the cranial end to the caudal end of the OPLL. The bilateral vertebral arteries were also marked in the same manner. After all ossification and vertebral arteries within the surgical area were marked on the axial views, they were reconstructed on the 3D image (Figure 2). The error in locating the points between the mapping software and the navigation system was 0.9 mm (0.7 and 0.6 mm in plane and depth, respectively) for 41 points that were measured preoperatively.

#### 2.3.4. Decompression and OPLL Floating Method with Microscopic AR Support

Corpectomy and OPLL floating were performed using a high-speed drill under a microscope with AR support, and the monitor was checked when necessary. For AR support, the head-up displays of the operating microscope (Opmi Pentero 900, Carl Zeiss Meditec, Oberkochen, Germany) were used (Figure 1C). A registration interface attached to the microscope allowed the tracking of its position. The reconstructed 3D image data were transferred and linked to the microscope to merge the live view and the AR microscopic view.

**Figure 3:**
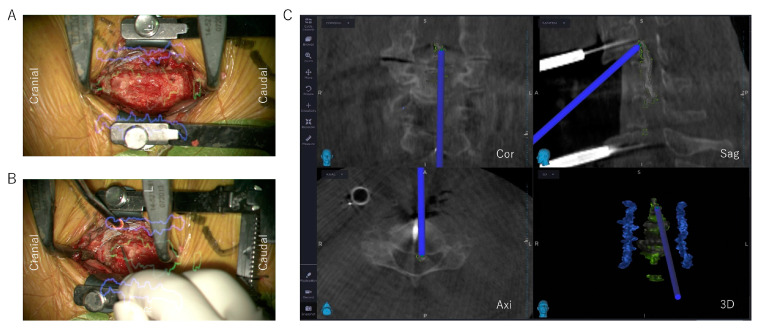
AR microscope view and navigation monitor of the ADF using the floating method. (**A**) Outline of the OPLL (yellow-green line) and vertebral arteries (blue line) visualized under the AR microscope view. (**B**) AR microscope view of a completed floating OPLL. The ossification edge (yellow-green outline in AR view) is completely decompressed, and the ossification is floating. (**C**) Navigation monitor after completed floating OPLL, with confirmation that the top ends of the ossification have been thinned and released. ADF, anterior decompression and fusion; AR, augmented reality; OPLL, ossification of the posterior longitudinal ligament.

The decompression area was determined with reference to the ossification outline visualized under the AR microscope view (Figure 3A). To avoid arterial injury, the locations of the bilateral vertebral arteries were also confirmed under the AR microscope view (Figure 3A). In addition to superimposing 3D objects in the operating microscope, the fused datasets were visualized in the navigation system. With microscopic AR support and a navigation system, the OPLL was ground as thin as possible using a drill, and the OPLL was then disconnected from the surrounding bone tissue at the top, bottom, left, and right edges of the OPLL. When the thinned OPLL resembled a board floating on water, the decompression of the entire spinal cord was complete (Figure 3B,C). The intraoperative O-arm imaging was sometimes taken again to confirm the final decompression. Subsequently, the cervical spine was reconstructed using an autologous fibula, a local bone in a metal cage, or a hydroxyapatite block with an anterior plate and screw system.

### 2.4. Clinical Assessments

Neurological status was assessed preoperatively and 1 year postoperatively using the Japanese Orthopaedic Association (JOA) scoring system. The JOA recovery rate was calculated as described by Hirabayashi et al. [28]. Additionally, the operation time, intraoperative bleeding, and intra-/postoperative complications were evaluated.

### 2.5. Radiographic Assessments

The lateral cervical spine radiographs of the neutral standing positions were obtained before and 1 year after surgery to measure the (1) C2–7 angle (C2–7 lordotic angle), (2) cervical sagittal vertical axis (SVA) (center of gravity of the head–C7 SVA), and (3) C7 slope [29]. Decompression was assessed using postoperative CT images. Postoperative CT images were taken within 3 months after surgery. Insufficient decompression widths in the axial or sagittal planes resulted in impingement between the OPLL and the surrounding bone tissue, inhibiting OPLL floatation [20]. Patients were followed postoperatively for at least 1 year.

### 2.6. Statistical Analysis

Neurological and radiographic assessments were performed by observers other than the principal surgeon. Statistical analyses were performed using the JMP software (version 14.2, SAS Institute, Cary, NC, USA). Intergroup comparisons were drawn using the χ^2^ test or the Mann–Whitney U test. A *p*-value of less than 0.05 was considered statistically significant.

## 3. Results

In total, 14 patients with cervical OPLL underwent ADF with microscopic AR support. The cohort included 4 women and 10 men with a mean age of 63.5 ± 10.9, and the mean BMI was 26.4 ± 5.1 kg/m^2^. The surgical procedure details are shown in Table 1. The operative time was 350.3 ± 99.2 min, and the intraoperative blood loss was 184.4 ± 211.2 g. A massive hemorrhage with bleeding > 1000 g was not observed in these cases. Intraoperative complications included cerebrospinal fluid (CSF) leakage in four (28.6 %) patients; however, they were managed well with the intraoperative local application of fibrin glue. No cases required additional surgery for the CSF fistula. One patient was managed with postoperative intubation because of upper airway obstruction but was weaned from the ventilator on the fifth postoperative day. Another patient developed postoperative C5 palsy, but motor strength was fully recovered 3 months after surgery. Neurological impairment was improved in all 14 patients, and the average improvement in the JOA score at 1 year postoperation was 47.6% ± 31.6% (preoperative JOA score: 11.7 ± 1.9; postoperative JOA score at 1 year: 14.3 ± 1.4). The average hospital stay was 27.7 ± 17.0 days.

The patient data for the AR-supported and non-AR-supported groups are summarized in Table 2. There were no significant differences in the age and sex ratios between the two groups. In addition, there were no significant differences in the C-JOA score recovery rate, operative time, and intraoperative blood loss between the two groups. However, there was no significant difference in the number of levels operated on between the two groups, but the number of corpectomies was significantly lower in the AR-supported group (*p* = 0.007). Regarding intra-/postoperative complications, there was no significant difference in the incidence of CSF leakage or nerve palsy between the two groups. Graft dislodgement was only observed in the non-AR-supported group. Reoperation due to inadequate decompression and graft dislodgement was only seen in the non-AR-supported group (10 cases, 18.9%). Impingement of floating OPLL was seen in one case in the AR-supported group, but after surgery the degree of spinal cord compression decreased, clinical symptoms improved, and no major complications requiring reoperation occurred in the AR-supported group. There was no difference in the postoperative cervical spine alignment (C2–7 angle, CSVA, and C7 slope) between the two groups.

### Case Presentation

**Figure 4:**
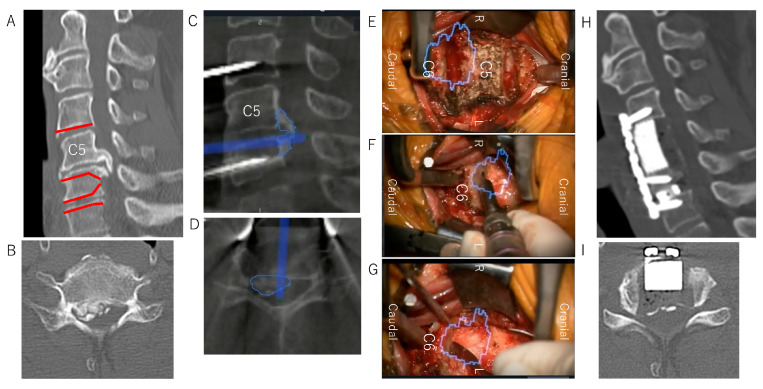
Case presentation. (**A**,**B**) Preoperative sagittal and axial CT views and the plan of the decompression line of the OPLL by undercutting cranially or caudally at the posterior aspect of the preserved vertebrae (red line). (**C**,**D**) Intraoperative sagittal and axial CT views of the OPLL, which were reconstructed after marking the outlines of ossification (blue line). (**E**–**G**) Intraoperative microscopic views with AR support and outlines of ossification (blue line): (**E**) before OPLL decompression, (**F**) during OPLL decompression, and (**G**) after OPLL floating. (**H**,**I**) Postoperative sagittal and axial CT views of sufficient OPLL floating. CT, computed tomography; OPLL, ossification of the posterior longitudinal ligament.

A 58-year-old man presented with bilateral hand clumsiness and gait disturbance. He underwent ADF using the floating method at the C4–C7 levels (Table 1, case No. 1). The decompression of the ossification at the C5/6 level required a C5 corpectomy and undercutting of the posterior aspect of the C6 vertebral body, with preservation of the majority of the superior endplate of C6 (Figure 4A,B). An intraoperative CT image was taken after the discectomy at the C4/5/6 level, and the ossification outline was reconstructed in 3D images (Figure 4C,D). The AR microscopic view enabled us to visualize the ossification outline behind the C6 vertebra, which could not be seen directly in the surgical field (Figure 4E). The edges of the ossification were thinned and cut along the outline of the ossification displayed on the AR microscopic view (Figure 4F). Finally, the thinned OPLL resembled a board floating on water, and the decompression of the entire spinal cord was complete (Figure 4G). The postoperative neurological symptoms improved well, and a postoperative CT showed sufficient decompression without any impingement of ossification (Figure 4H,I).

## 4. Discussion

To our knowledge, this is the first report on the introduction of microscopic AR into ADF using the floating method for the treatment of cervical OPLL. The microscopic AR support is considered to be effective because the shape of ossification, which is not directly visible intraoperatively, can be visualized through a microscope, which enables sufficient decompression of the OPLL, even when the number of corpectomies is reduced. In addition, reoperations due to major complications such as decompression failure and graft dislocation could be eliminated.

AR technology has been introduced into surgery since the 1990s. Franken et al. explored a three-dimensional on-screen microsurgical system (TOMS) that eliminated the need to view the operative field through microscope eyepieces [30]. In the past few years, AR technology has been used in urologic and neurosurgical surgeries to view live endoscopic images and intraoperative navigation screens [31]. Justin et al. introduced AR technology to 79 patients undergoing neurosurgery and reported that AR technology can be safely used for various vascular and oncologic intracranial pathologies [26]. In this study, AR technology was applied to ADF using the floating method for cervical OPLL because we believe that AR technology can make surgery much safer by facilitating the intraoperative anatomical orientation and identification of OPLL.

In recent years, AR technology has been employed in spine surgery. AR guidance in spine surgery enables the accurate placement of instruments such as pedicle screw insertion, rod bending, and percutaneous vertebroplasty [25,32]. Furthermore, the accurate placement of instruments with AR guidance was reported to reduce the operative time [33,34]. Molina et al. applied AR technology to en bloc lumbar osteotomy and reported a superior understanding of the 3D anatomy, thereby facilitating surgery [35]. Recent advances in AR have opened the possibility of using these technologies for navigation by allowing precise optical visibility via a head-mounted display (HMD). However, HMDs are special devices, and their use is limited to surgery at each facility, so their versatility is still limited. On the other hand, microscopic AR support can be used by linking navigation systems and microscopes at each facility, making it less costly and more versatile than HMDs. Carl et al. used microscopic AR support in spine tumor surgery with corpectomy [24]. A microscopic AR display demonstrated the close matching of the outline of the tumor and the outline of the vertebral body replacement and its augmented representation. Umebayashi et al. applied AR technology to keyhole surgery. A case of transvertebral anterior cervical foraminotomy and a case of posterior foraminotomy displayed the AR imaging model on the surgical microscope view [36]. In the keyhole surgery, full anatomic exposure to identify relevant landmarks is inherently not attempted. Displaying landmarks in the microscopic field of view may be significantly beneficial. Sommer et al. applied AR technology to MISTLIF and displayed the AR imaging landmark on the surgical microscope view, which was useful in minimally invasive surgery, where it was difficult to fully grasp the entire surgical site [37]. Microscopic AR support could help to identify the correct anatomical landmarks by highlighting them in patients with severe degenerative changes and complicated anatomical conditions. Additionally, the increased ease of finding the anatomical landmarks could help surgeons by making surgery less demanding and minimizing surgeon errors. Consistent with previous reports of AR in spine surgeries, it enabled us to better understand the anatomical orientation in our application of AR to ADF using the floating method.

ADF for cervical OPLL is an ideal surgical technique for the direct decompression of the spinal cord, where the ossification is floated or resected [7,8,9,10,11,12,13,14]. Posterior decompression techniques, such as laminoplasty (LAMP), are relatively simple procedures for the indirect decompression of the spinal cord. In a nonrandomized prospective study comparing the two surgical techniques, ADF was shown to have better outcomes than LAMP in patients with kyphosis or high ossification occupancy in the spinal canal (>50%) [15]. In a systematic review comparing the two surgical techniques to assess improvements in neurological symptoms, ADF was superior to LAMP, especially in patients with high intracanalicular occupancy of OPLL (>60%) [17]. Furthermore, LAMP is not suitable for patients with cervical myelopathy caused by OPLL who have high CSVA (center of gravity of the head) alignment (≥40 mm), even in cases without massive OPLL or kyphotic alignment [16]. Therefore, surgical procedures for cervical myelopathy caused by OPLL are often chosen based on the OPLL size and cervical spine alignment, and LAMP may be insufficient for patients with massive OPLL and/or kyphotic alignment [18]. Although ADF using the floating method for cervical OPLL has been performed for a long time, due to the high procedural difficulty of ADF using the floating method, it is sometimes difficult to accurately identify the full shape of OPLL, which exists behind the vertebrae, mixed with ligamentous or cartilaginous tissues [9,19]. Studies have reported that increasing the number of corpectomies for sufficient spinal cord decompression increases the frequency of implant complications [19,38]. Therefore, as demonstrated in the case presentation, reducing the number of corpectomies by undercutting cranially or caudally at the posterior aspect of the preserved vertebrae was reasonable to minimize the risk of reconstruction failure. However, a “selective wedge corpectomy” is considered to be further technically demanding and requires experienced decompression skills and perfect recognition of the location and shape of the OPLL.

Recent advancements in intraoperative imaging modalities, such as mobile CT and navigation systems, and their application to ADF using the floating method for OPLL enable surgeons to easily identify the ossification outline during an operation. Because of these technological advances, insufficient decompression caused by the impingement of ossification can be reduced, improving clinical outcomes [20,22]. Furthermore, as shown in the case presentation, ADF with AR support has made it possible to more directly understand the ossification shape in the microscopic view without checking the navigation screen. ADF with microscopic AR support was well suited for OPLL decompression, especially for cases that required oblique undercutting of the posterior aspect of the vertebral body, and allowed sufficient decompression of the ossification while preserving the vertebral body. In addition, “selective wedge corpectomy” with AR support allowed sufficient decompression with a reduced number of corpectomies, suggesting the possibility of preventing reoperation due to postoperative graft dislodgement.

In this study, ADF using the floating method with AR support was performed in 14 patients, and favorable postoperative results were obtained. Based on the improvement in the JOA score (52.5%–71.4%), operative time (271.2–335.9 min), and blood loss (287.5–600 g) values reported in previous multicenter studies, our surgical outcomes with AR support were comparable with previous reports [11,12,13,19,22,38,39,40,41]. In addition, ADF using the floating method with AR support did not cause serious complications such as major intraoperative bleeding and reoperation. Thus, ADF with AR support may improve postoperative clinical outcomes by reducing intraoperative complications, such as insufficient decompression and vascular injuries, by projecting virtual anatomic images into the operator’s view in real time.

The practical applications of ADF using the floating method with AR support are numerous and significant. One of the main advantages of AR support is the ability to accurately identify the shape and location of OPLL during surgery. This can help reduce the risk of insufficient decompression and improve surgical outcomes. By overlaying preoperative images onto the surgical field, AR provides real-time visualization of the shape and location of OPLL, allowing surgeons to make more precise and informed decisions during the procedure. AR also enhances the visualization of anatomical structures, such as nerves and blood vessels, which can be difficult to identify during surgery. In addition, AR support for ADF using the floating method allows for “selective wedge corpectomy” and reduces the number of corpectomies that are needed, as it allows the visualization of ossification behind the vertebral body, which is not visible. Furthermore, AR support for ADF using the floating method has been shown to reduce the need for reoperation due to postoperative graft dislodgement or insufficient decompression. This is a significant advantage because additional surgeries increase the risk of major complications and can negatively impact patient outcomes. Therefore, although complications have occurred in some cases, the potential benefits of AR navigation in reducing the need for reoperation and improving intraoperative accuracy and safety should be recognized.

This study has two main limitations, its retrospective nature and the small sample size, which may affect the strength of our conclusions and the generalizability of our findings. The limited case series is due to the fact that only a few centers have access to AR support technology. Despite these limitations, our study provides valuable insights into the potential benefits of using AR support in surgical contexts. We hope our research serves as a basis for future large-scale prospective studies that can further validate and strengthen the evidence in this area. Ultimately, we believe that our results will contribute to the development of the ADF surgical floating and decompression technique for cervical OPLL and hold clinical significance.

## 5. Conclusions

This is the first report on the introduction of microscopic AR into ADF using the floating method for the treatment of cervical OPLL. AR support for ADF using the floating method had favorable clinical results and enabled sufficient decompression of OPLL.

## Figures and Tables

**Table 1:** Patient characteristics and surgical procedure details.

Case No.	Age (years)	Sex (M/F)	Procedure of ADF Using the Floating Method	Operative Time (min)	Blood Loss (grams)	Intraoperative Complication	Perioperative Complication
1	58	M	C4–6ACCF C6/7ACDF	292	30		Intubation management (postoperative days 1–4)
2	54	M	C3–5ACCF	390	80	CSF leakage	
3	54	M	C3/4AF C4/5/6/7ACDF	312	35		
4	63	M	C4–6ACCF C6/7ACDF	263	35		
5	69	F	C4/5ACDF C5–7ADF	240	400		
6	63	M	C2–5ACCF C5/6ACDF	461	29		
7	78	F	C3–6ACCF	313	500	CSF leakage	
8	55	M	C3/4ACDF C4–7ACCF	476	56	CSF leakage	
9	84	M	C3/4/5ACDF	186	10		
10	75	M	C4–7ACCF	270	100		
11	63	F	C3/4ACDF C4–7ACCF	518	372		
12	54	M	C3/4ACDF C4–7ACCF	444	630		
13	46	F	C4–7ACCF	394	295	CSF leakage	C5 palsy
14	72	M	C4/5ACDF C5–7ACCF	345	10		

ADF: anterior decompression and fusion; ACCF: anterior cervical corpectomy and fusion; ACDF: anterior cervical discectomy and fusion; AF: anterior fusion; CSF: cerebrospinal fluid.

**Table 2:** Univariate analysis of the patients in the non-AR-supported and AR-supported groups.

	Non-AR (n = 53)	AR (n = 14)	*p*
Clinical outcomes			
Age (years)	63.4 ± 8.7	63.5 ± 10.9	0.99
Sex (M/F, M%)	45/8 (84.9%)	10/4 (71.4%)	0.25
Pre JOA (score)	10.4 ± 2.4	11.7 ± 1.9	0.06
Post JOA (score)	14.1 ± 2.1	14.3 ± 1.4	0.74
Recovery rate (%)	56.2 ± 27.0	47.6 ± 31.6	0.64
Operative outcomes			
Number of operated levels	3.2 ± 0.8	3.3 ± 0.7	0.69
Number of corpectomies	2.0 ± 0.7	1.4 ± 0.7	0.007 *
Operative time (min)	295.6 ± 93.5	350.3 ± 99.2	0.071
Intraoperative blood loss (g)	302.1 ± 895.7	184.4 ± 211.2	0.63
CSF leakage, n (%)	8 (15.1%)	4 (28.6%)	0.19
Nerve palsy, n (%)	10 (18.9%)	1 (7.1%)	0.48
VA injury, n (%)	0 (0%)	0 (0%)	-
Impingement of floating OPLL, n (%)	7 (13.2%)	1 (7.1%)	0.47
Graft dislodgement, n (%)	9 (17.0%)	0 (0%)	0.18
Reoperation, n (%)	10 (18.9%)	0 (0%)	0.078
Radiographic outcomes			
Pre C2–7 angle (°)	5.4 ± 12.7	1.0 ± 11.1	0.091
Pre CSVA (mm)	30.5 ± 20.3	24.6 ± 14.4	0.31
Pre C7 slope (°)	26.9 ± 8.6	21.8 ± 2.3	0.054
Post C2–7 angle (°)	7.9 ± 9.9	4.1 ± 10.6	0.22
Post CSVA (mm)	25.7 ± 18.7	23.9 ± 13.5	0.74
Post C7 slope (°)	25.0 ± 1.2	20.2 ± 2.3	0.091

JOA, Japanese Orthopaedic Association for cervical myelopathy; Pre, preoperative; Post, postoperative; CSF, cerebrospinal fluid; VA, vertebral artery; CSVA, cervical sagittal vertical axis. Data are shown as means (standard deviations) unless otherwise indicated. *p* < 0.05 was considered statistically significant. * *p* < 0.05.

## Data Availability

Detailed data are available from the first author upon request.
